# Supplementary material for: Global crop yields can be lifted by timely adaptation of growing periods to climate change
Source: Nat Commun. 2022 Nov 18;13:7079. doi: 10.1038/s41467-022-34411-5 (PMC9674574; doi:10.1038/s41467-022-34411-5)
Supplement: Supplementary file 1 — Supplementary Information [file 41467_2022_34411_MOESM1_ESM.pdf]

Supplementary Information

Global crop productivity can be lifted by timely adaptation of phenology to climate change

Sara Minoli*, Jonas Jägermeyr, Senthold Asseng, Anton Urfels, Christoph Müller

*Corresponding author. Email: sara.minoli@pik-potsdam.de

Contents:

Supplementary Figures 1 to 25
Supplementary Tables 1 to 3
Supplementary References

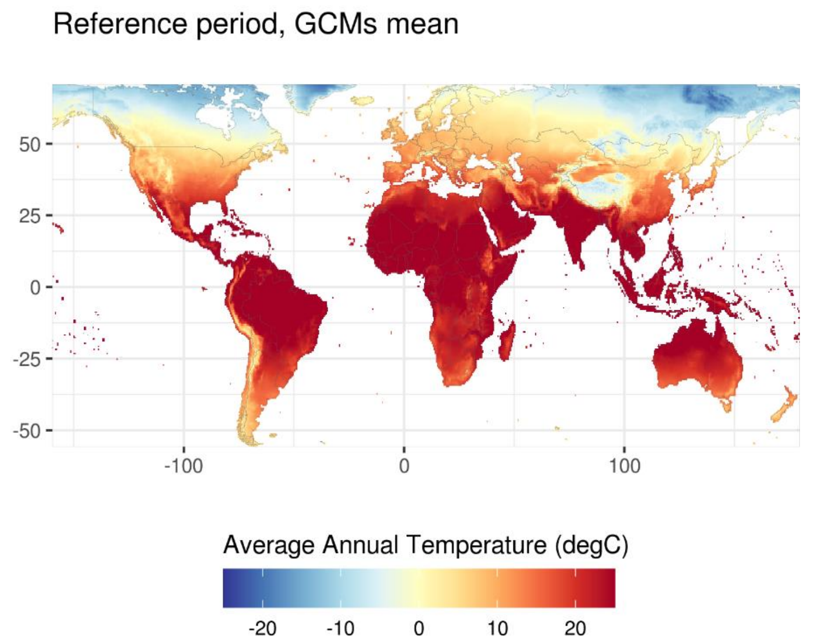

Supplementary Figure 1. Temperature in the reference period. Average annual mean air temperature (°C) over land for the simulation reference period (1986-2005), shown as the mean across four GCMs (GFDL-ESM2M, HadGEM2-ES, IPSL-CM5A-LR and MIROC5)[1].

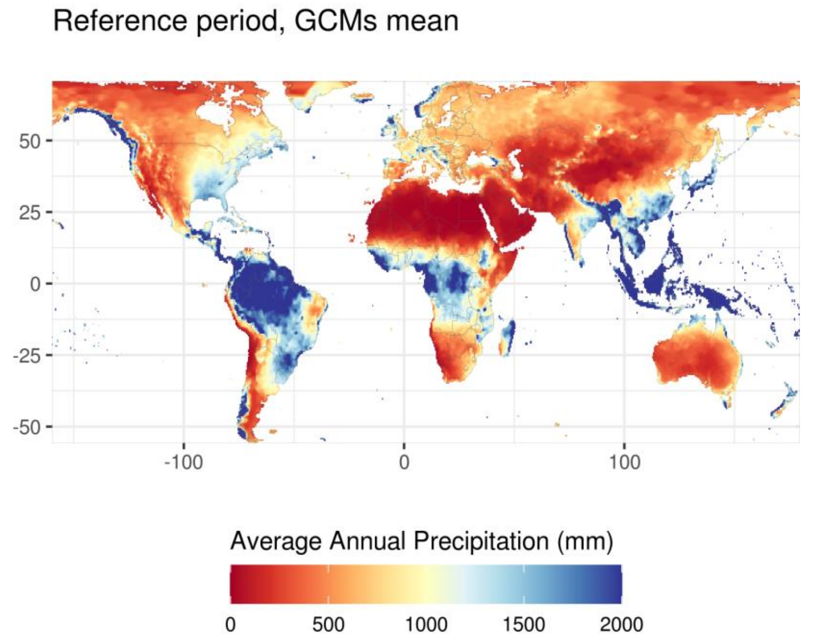

Supplementary Figure 2. Precipitation in the reference period. Average annual precipitation (mm) over the land for the simulation reference period (1986-2005), shown as the mean across four GCMs (GFDL-ESM2M, HadGEM2-ES, IPSL-CM5A-LR and MIROC5)[1].

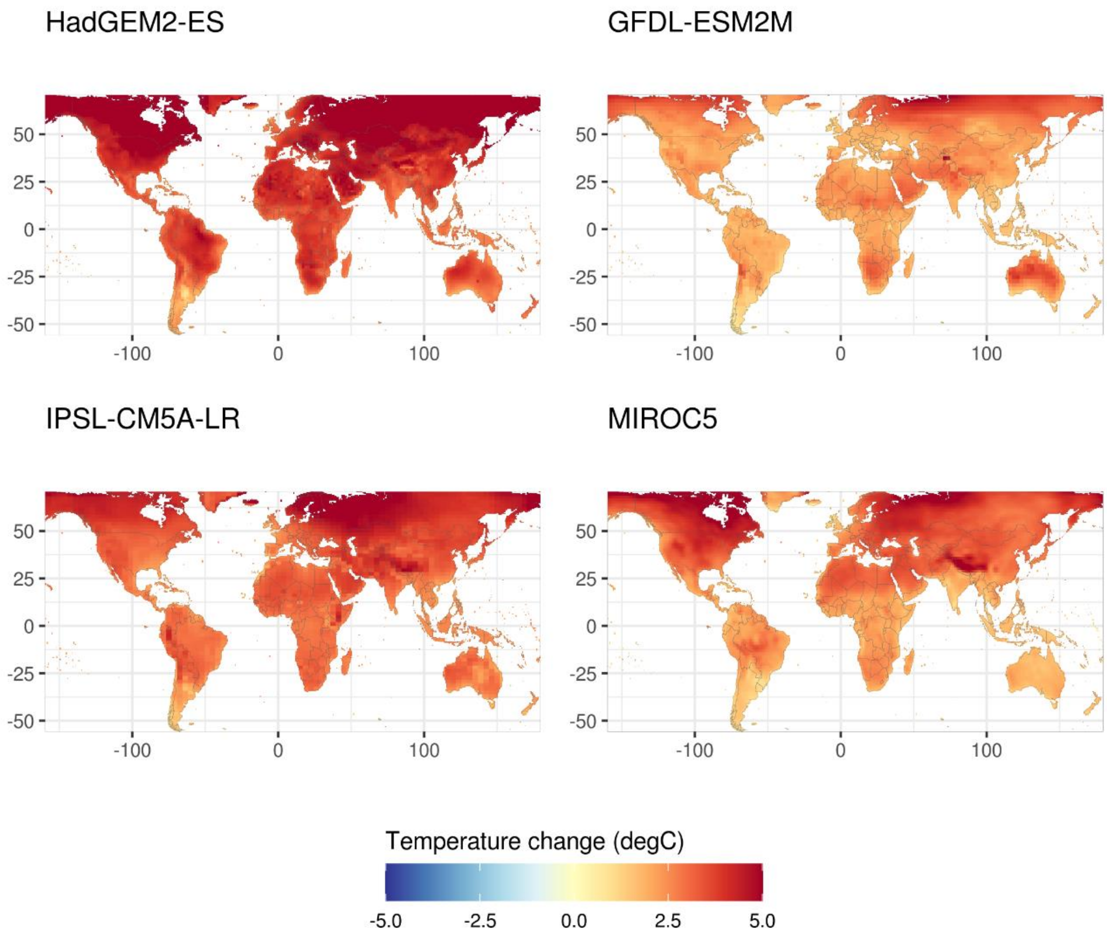

Supplementary Figure 3. Future temperature changes. Changes in annual mean temperature (°C) at the end of the century (2080-2099) compared to the reference period (1986-2005) according to four GCMs projections (GFDL-ESM2M, HadGEM2-ES, IPSL-CM5A-LR and MIROC5) under RCP6.0[1].

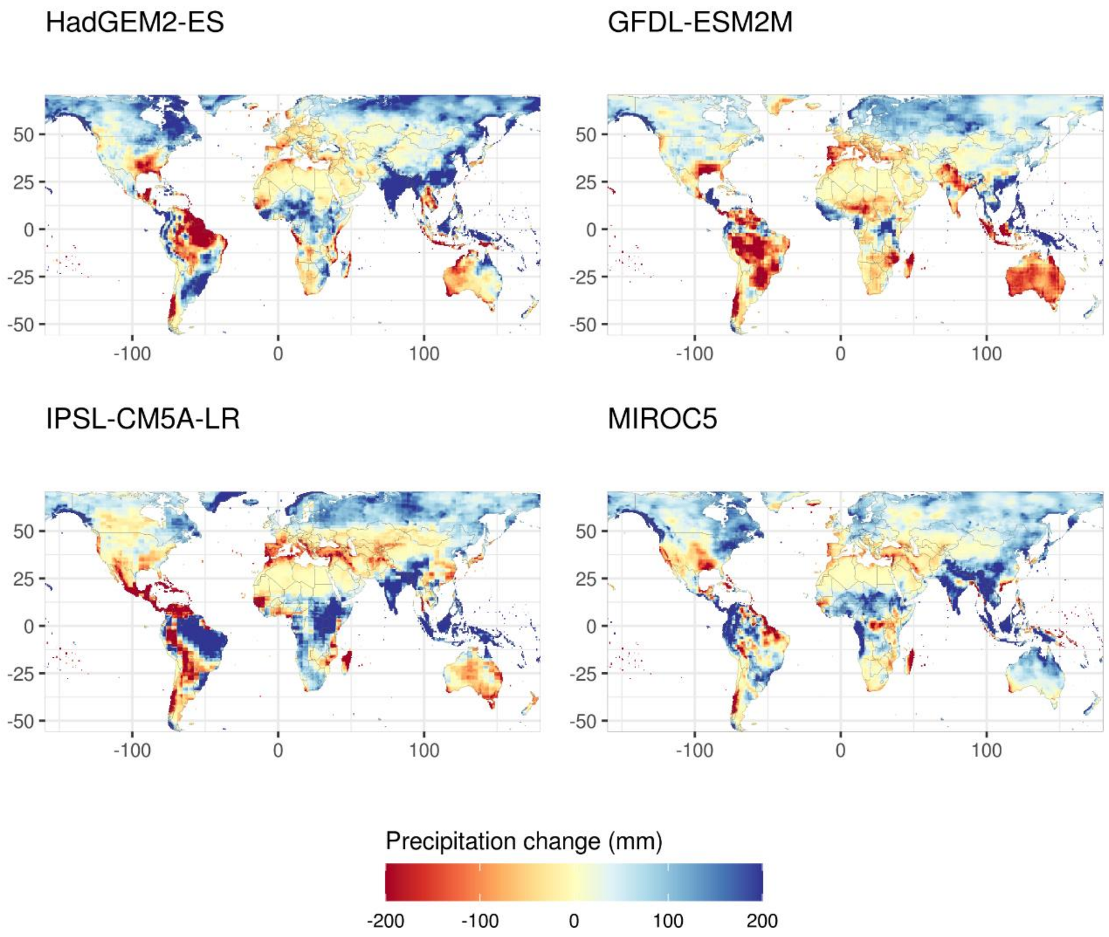

Supplementary Figure 4. Future precipitation changes. Changes in annual cumulative precipitation (mm) at the end of the century (2080-2099) compared to the reference period (1986-2005) according to four GCMs projections (GFDL-ESM2M, HadGEM2-ES, IPSL-CM5A-LR and MIROC5) under RCP6.0[1].

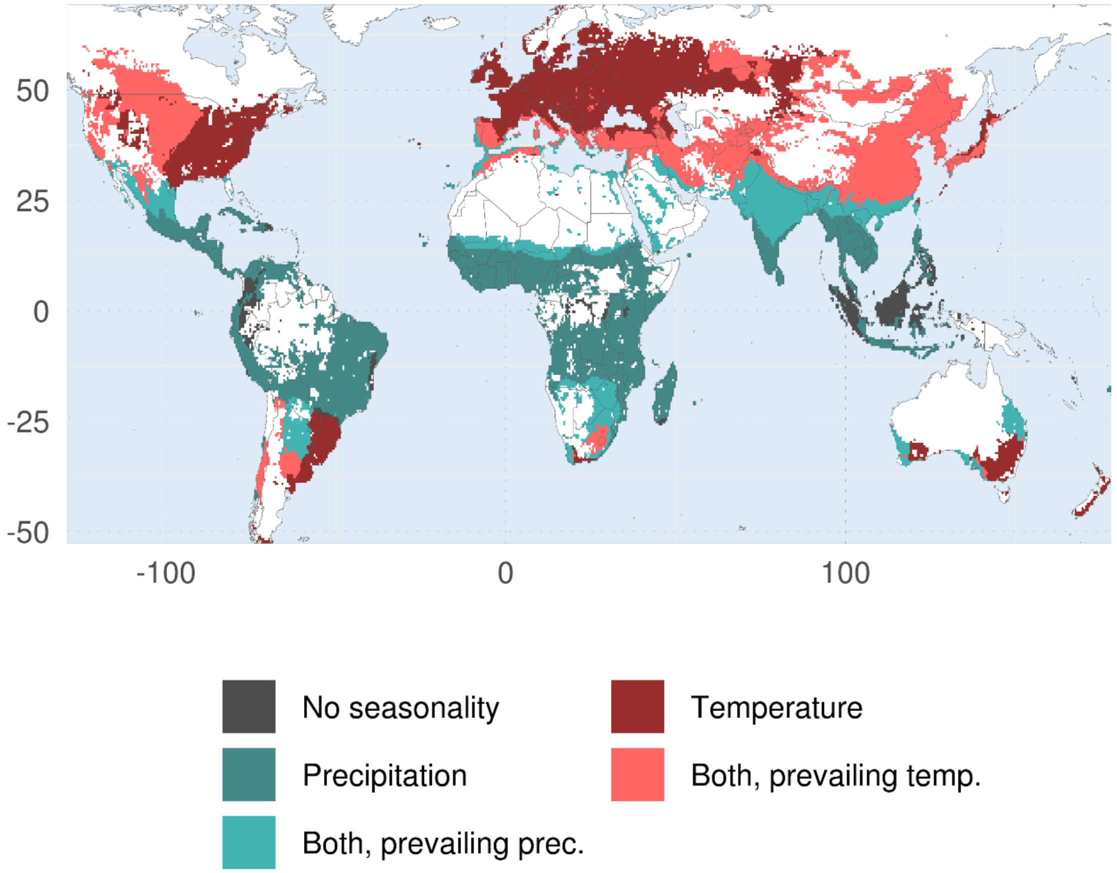

Supplementary Figure 5. Climate seasonality types driving the simulated sowing dates. Climate seasonality represents the average profiles of temperature and precipitation. The prevailing seasonality type determines whether computed sowing dates are driven by temperature or precipitation. The seasonality classification (*No seasonality*; *Precipitation*; *Both, prevailing precipitation*; *Both, prevailing temperature*; *Temperature*) is based on the coefficient of variation of monthly values of temperature and precipitation (20-years average)[2].Note that winter wheat sowing dates are driven by temperature seasonality only and cannot be grown in *No seasonality* climatic regions.

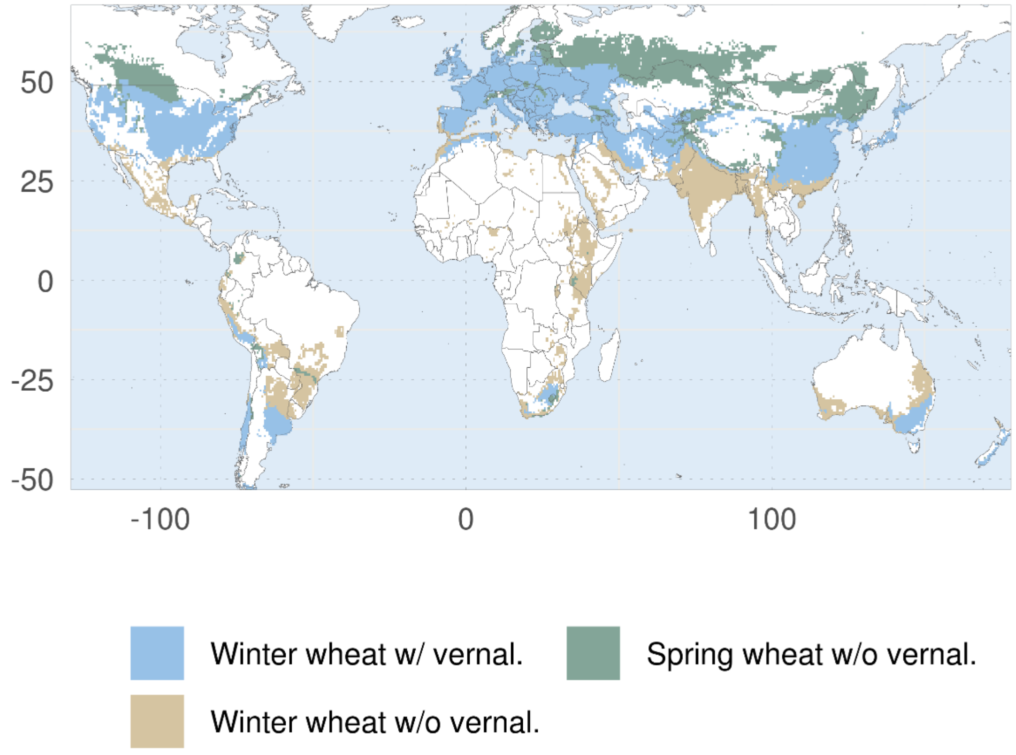

Supplementary Figure 6. Wheat types classification and distribution. Types of wheat simulated by the rule-based crop calendar model: *winter wheat with vernalization*, represents wheat cultivars with vernalization requirements that are sown in fall and grown over cold winters; *winter wheat without vernalization*, represents wheat cultivars without vernalization requirements that are sown in fall and grown over mild winters; *spring wheat without vernalization*, represents wheat cultivars without vernalization requirements that are sown in spring. The patterns show the wheat types distribution as computed by the rule-base model for the historical period driven by the observation-based reanalysis climate dataset WFDEI[3].

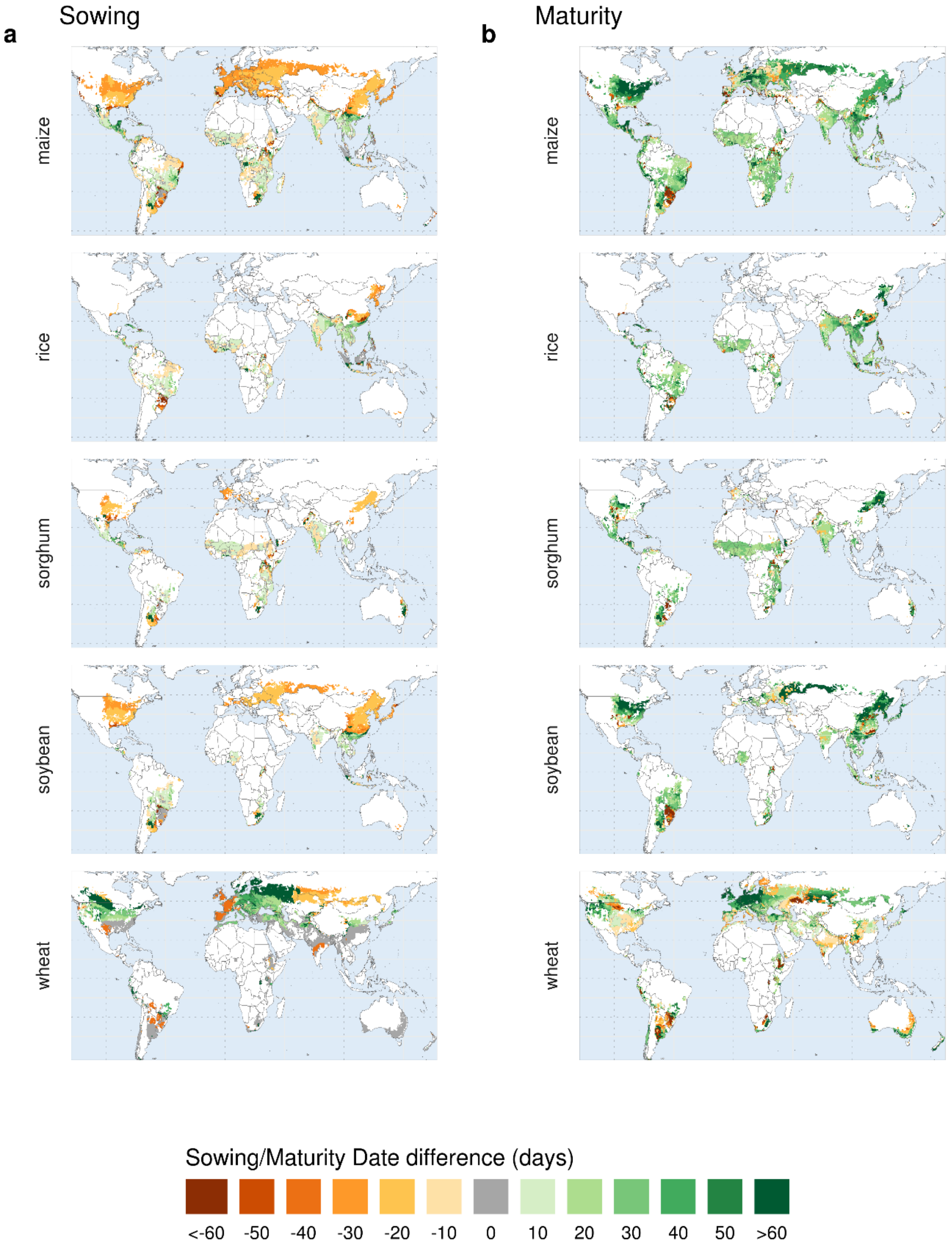

Supplementary Figure 7. Crop calendars adaptation. Differences (days) in simulated average sowing (A) and maturity (B) dates between *timely adaptation* and *no adaptation* scenarios for the same climate period (2080-2099, RCP 6.0). Crop calendars are climate-scenario (GCM) specific, here the changes are reported for the HadGEM2-ES climate scenario.

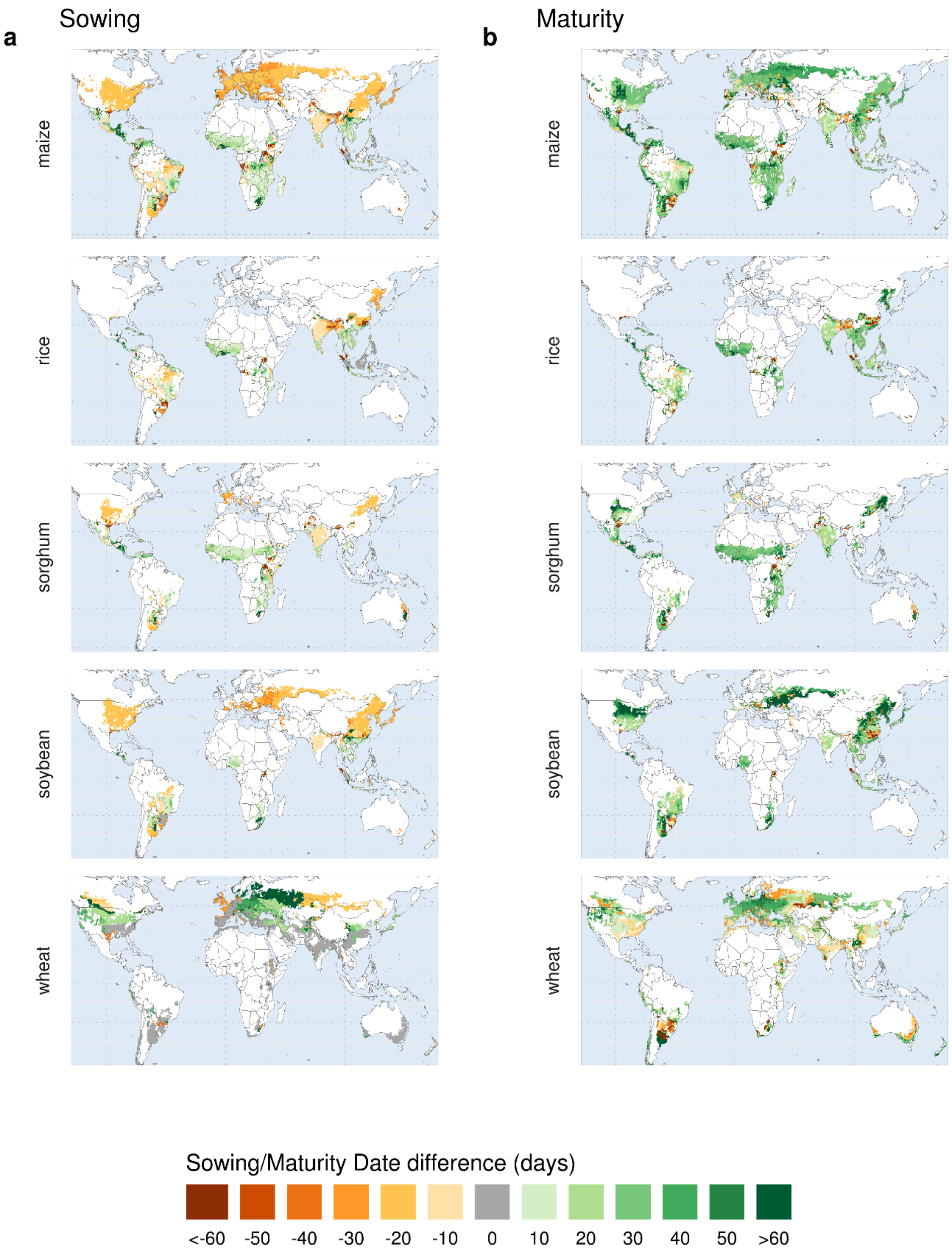

Supplementary Figure 8. Crop calendars adaptation. Differences (days) in simulated average sowing (A) and maturity (B) dates between *timely adaptation* and *no adaptation* scenarios for the same climate period (2080-2099, RCP 6.0). Crop calendars are climate-scenario (GCM) specific, here the changes are reported for the IPSL-CM5A-LR climate scenario.

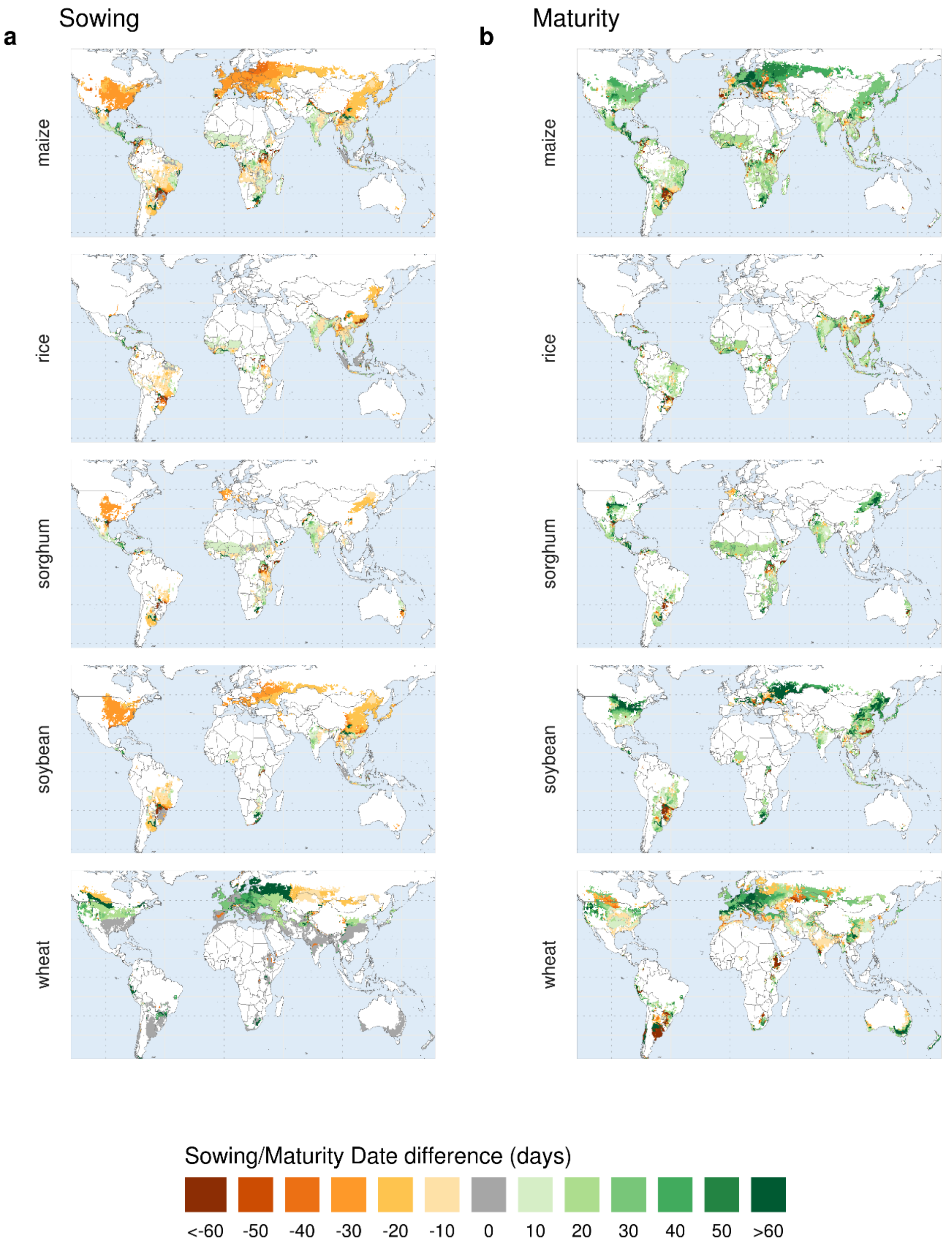

Supplementary Figure 9. Crop calendars adaptation. Differences (days) in simulated average sowing (A) and maturity (B) dates between *timely adaptation* and *no adaptation* scenarios for the same climate period (2080-2099, RCP 6.0). Crop calendars are climate-scenario (GCM) specific, here the changes are reported for the MIROC5 climate scenario.

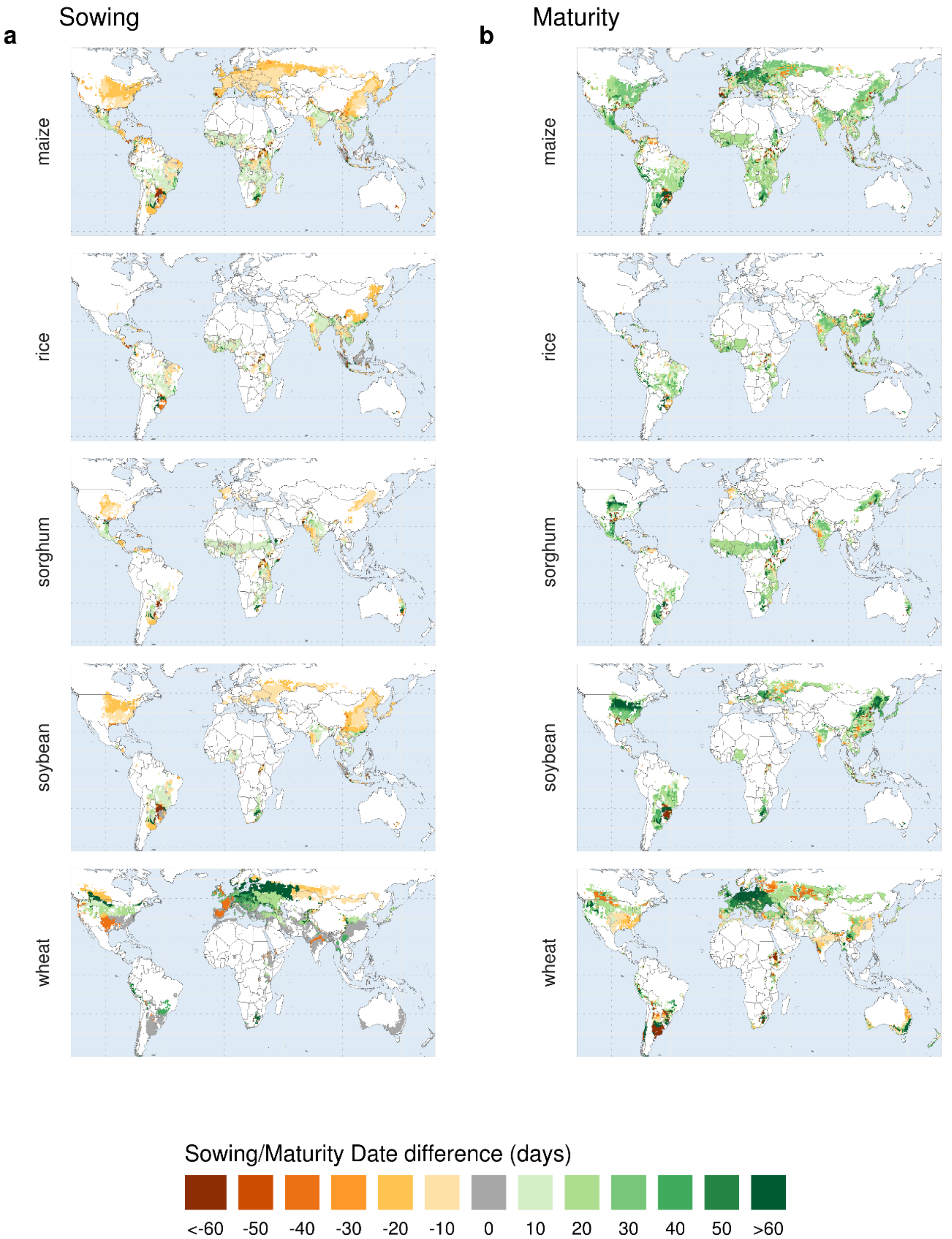

Supplementary Figure 10. Crop calendars adaptation. Differences (days) in simulated average sowing (A) and maturity (B) dates between *timely adaptation* and *no adaptation* scenarios for the same climate period (2080-2099, RCP 6.0). Crop calendars are climate-scenario (GCM) specific, here the changes are reported for the GFDL-ESM2M climate scenario.

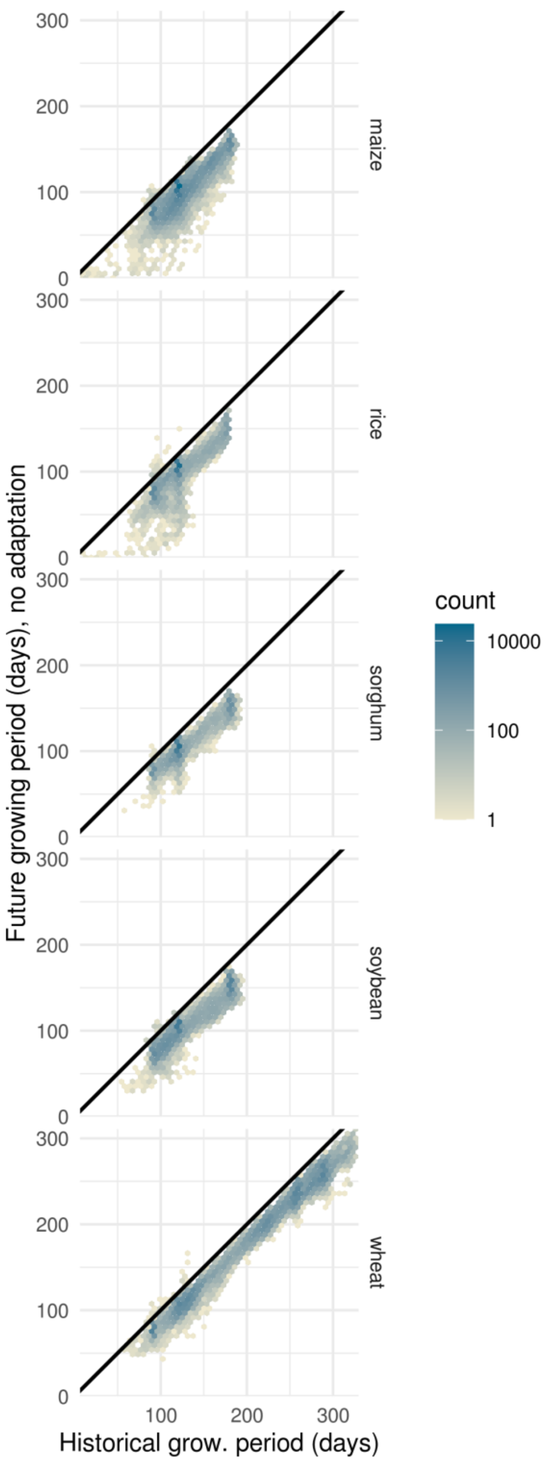

Supplementary Figure 11. Climate change impact on growing period without adaptation. Grid-cell level comparison of future (2080-2099) against reference (1986-2005) growing period duration (days) per crop under the *no adaptation* scenario. The colour scale indicates the number of grid-cells included in each displayed dot.

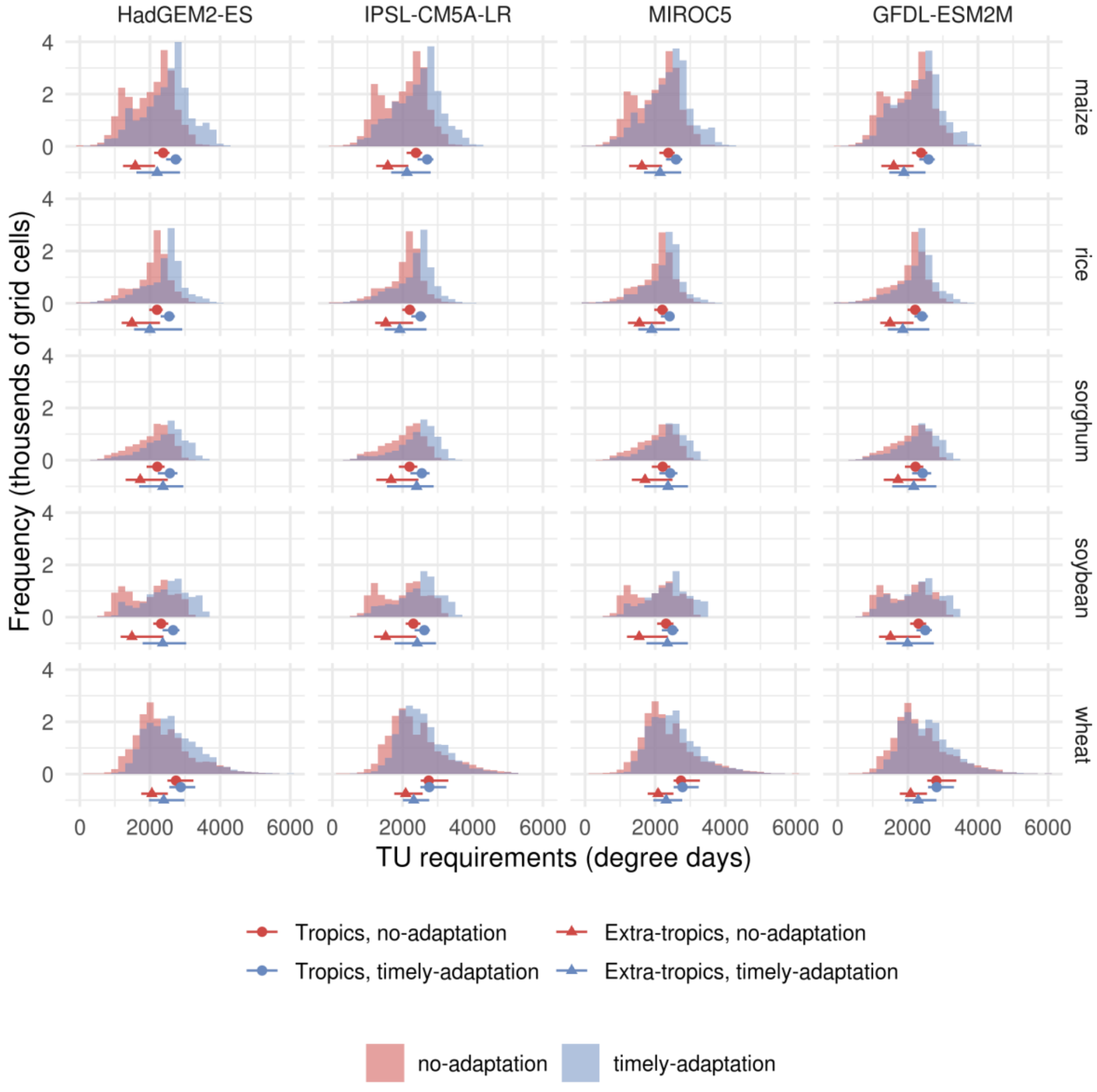

Supplementary Figure 12. Cultivars required for adaptation. Distributions of thermal unit requirements (TU_{req}) of the cultivars adapted to the historical (*no adaptation*) and future (*timely adaptation*) climate, displayed per crop and GCM.

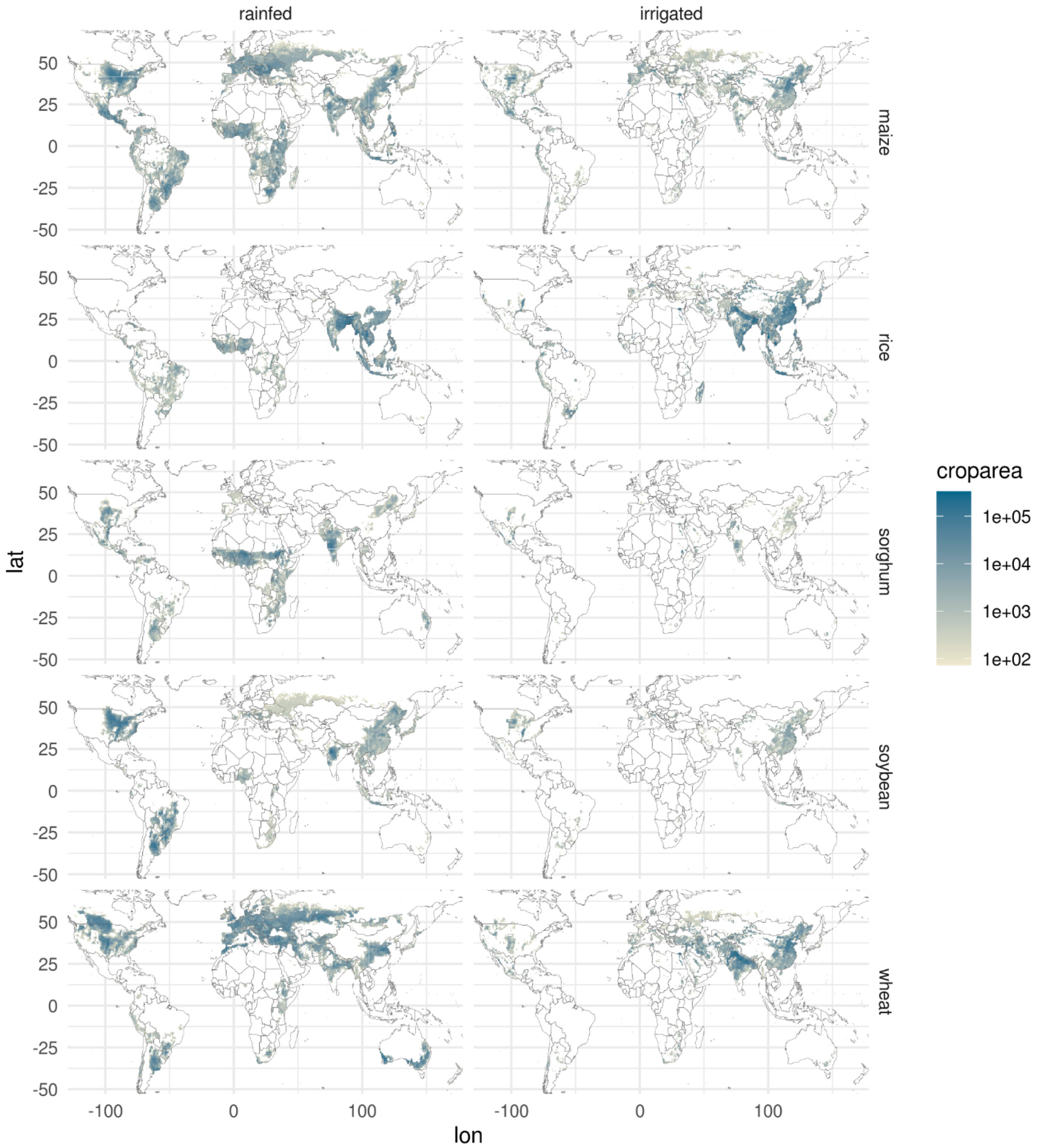

Supplementary Figure 13. Cropland area. Grid-cell,crop- and irrigation-specific area (ha) as reported by the MIRCA2000[4] dataset, used in this study for calculating area-weighted yields

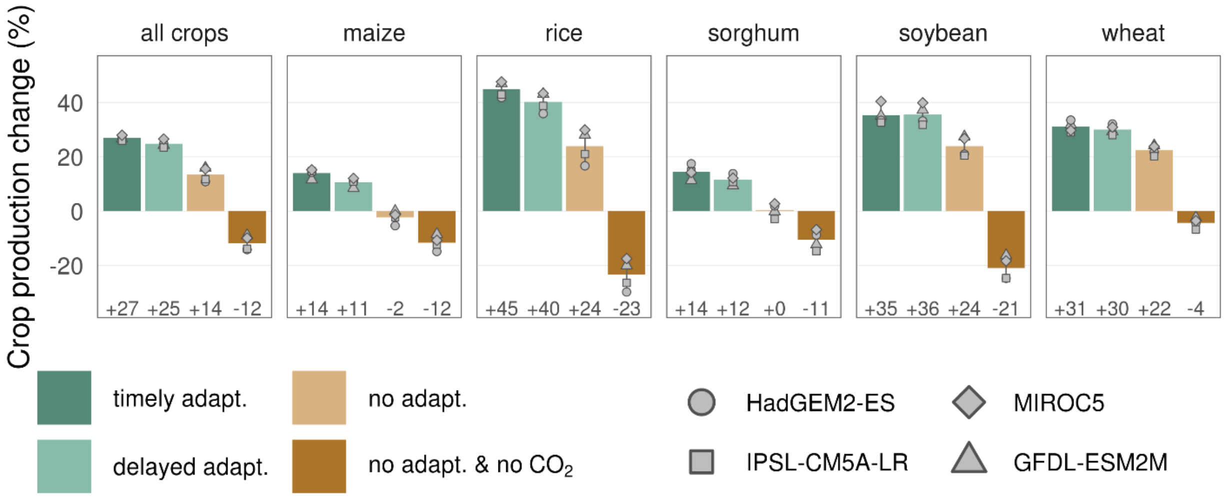

Supplementary Figure 14. Global responses of crop yields to climate change and adaptation. Crop yield changes are calculated as the difference between the future (2080-2099) and the reference (1986-2005) periods. *Timely adaptation*, *delayed adaptation* and *no adaptation* refer to different adaptation scenarios forced by RCP6.0 climate; *no adaptation & no* CO_2 refer to a *no adaptation* scenario, with climate change but without atmospheric CO_2 increase. Yields of individual crops is computed as the area-weighted mean of yields in all grid cells growing that crop. Responses are shown for all crops aggregated as well as for individual crops. Bars represent the mean across GCMs (n = 4 GCMs), whiskers display the range across GCMs, and grey symbols refer to individual GCMs.

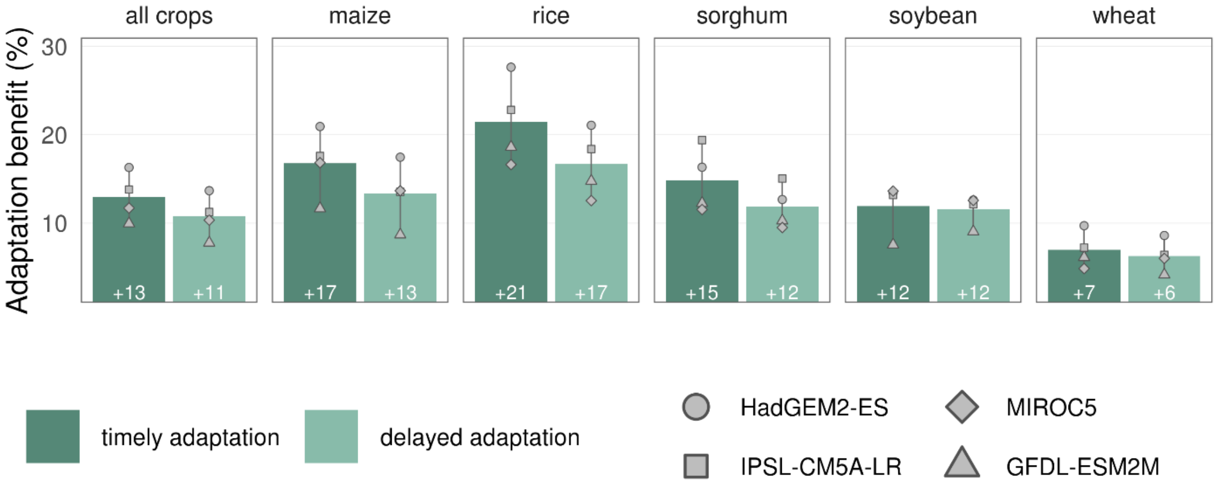

Supplementary Figure 15. Benefits of sowing and cultivar adaptation on global crop yields (2080-2099, RCP 6.0, no CO_2). Benefits on global yields are reported for all crops aggregated and for each individual crop, along with the uncertainty under different climate scenarios, without atmospheric CO_2 increase. The four adaptation scenarios indicate different levels of adaptation (adapt.): *timely adaptation*, sowing dates and cultivars adapted as the climate is changing (2080-2099); *cultivar adaptation*, sowing fixed at the reference level, only cultivars adapted as in timely adaptation; *sowing date adaptation*, only sowing dates adapted as in timely adaptation, cultivars fixed at the reference level; *delayed adaptation* both sowing dates and cultivar adapted but with 20-years delay, to 2060-2079 climate. The global yield of an individual crop is computed as the area-weighted mean yield across all grid cells growing that crop. In grid cells where adaptation of growing periods returned either no benefit or maladaptation (yield difference is equal or larger zero) yield losses were considered equal zero. Bars represent the mean across GCMs (n = 4 GCMs), whiskers display the range across GCMs, and grey symbols refer to individual GCMs.

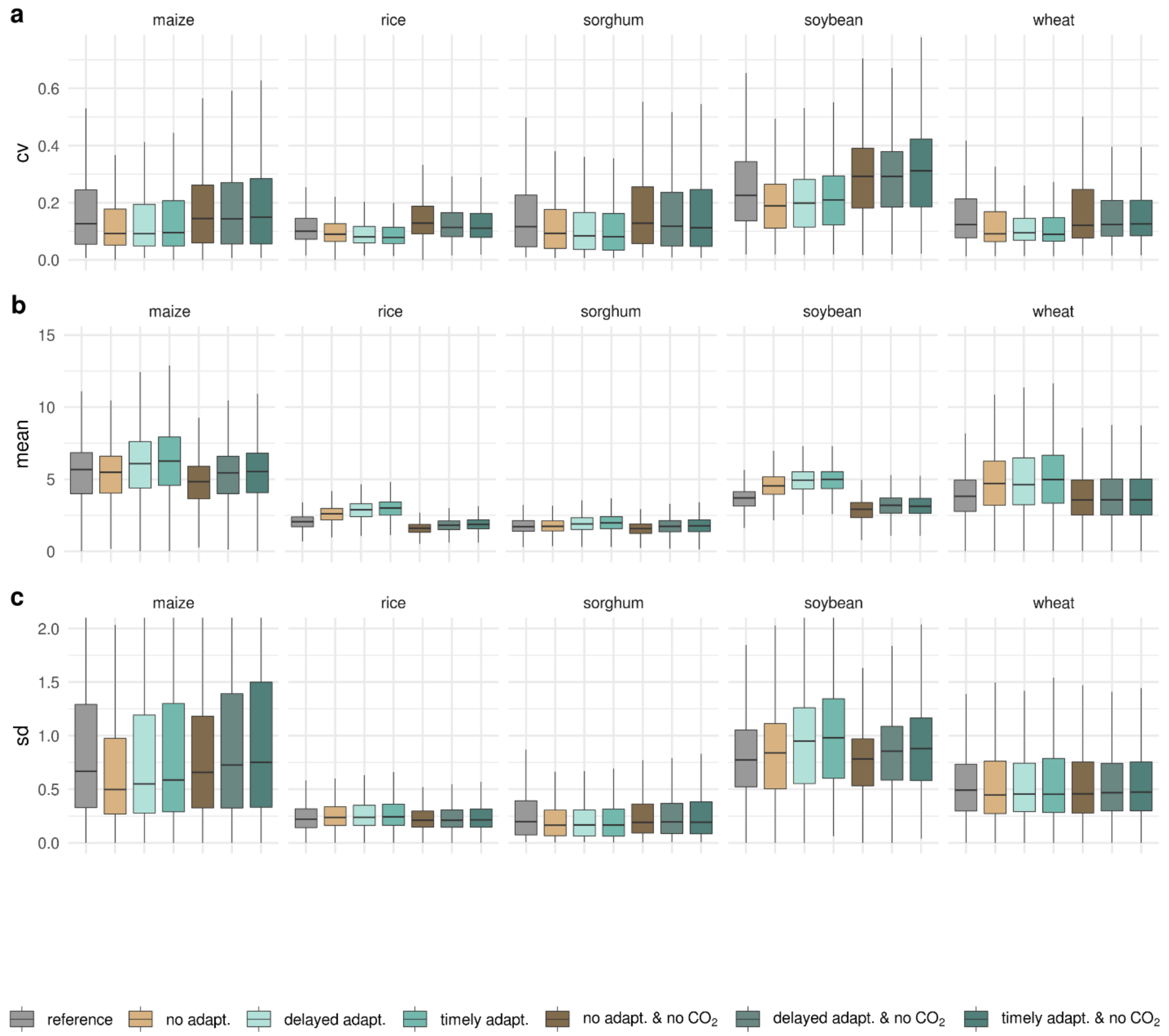

Supplementary Figure 16. Yield variability under increasing atmospheric CO_2, climate change and adaptation. Interannual yield variability measured as (A) coefficient of variation (cv) and its components, (B) mean yield and (C) standard deviation (sd). Scenarios with climate change but without atmospheric CO_2 increase are indicated as *no CO_2*. The coefficient of variation is computed as year-to-year yield deviation normalized by the mean yield over the same period (1986-2005 for the *reference* scenario and 2080-2099 for all other scenarios). Each box plot shows the distribution across all area-weighted grid-cell value of the respective metric with n (maize) = 655606; n (rice) = 316190; n (sorghum) = 227948; n (soybean) = 296499; n (wheat) = 537670. The centre of the box represents the median, the edges of the box the 25th and 75th percentile, and the whiskers the 1.5 interquartile range (1.5IQR). Values outside the 1.5IQR are not shown

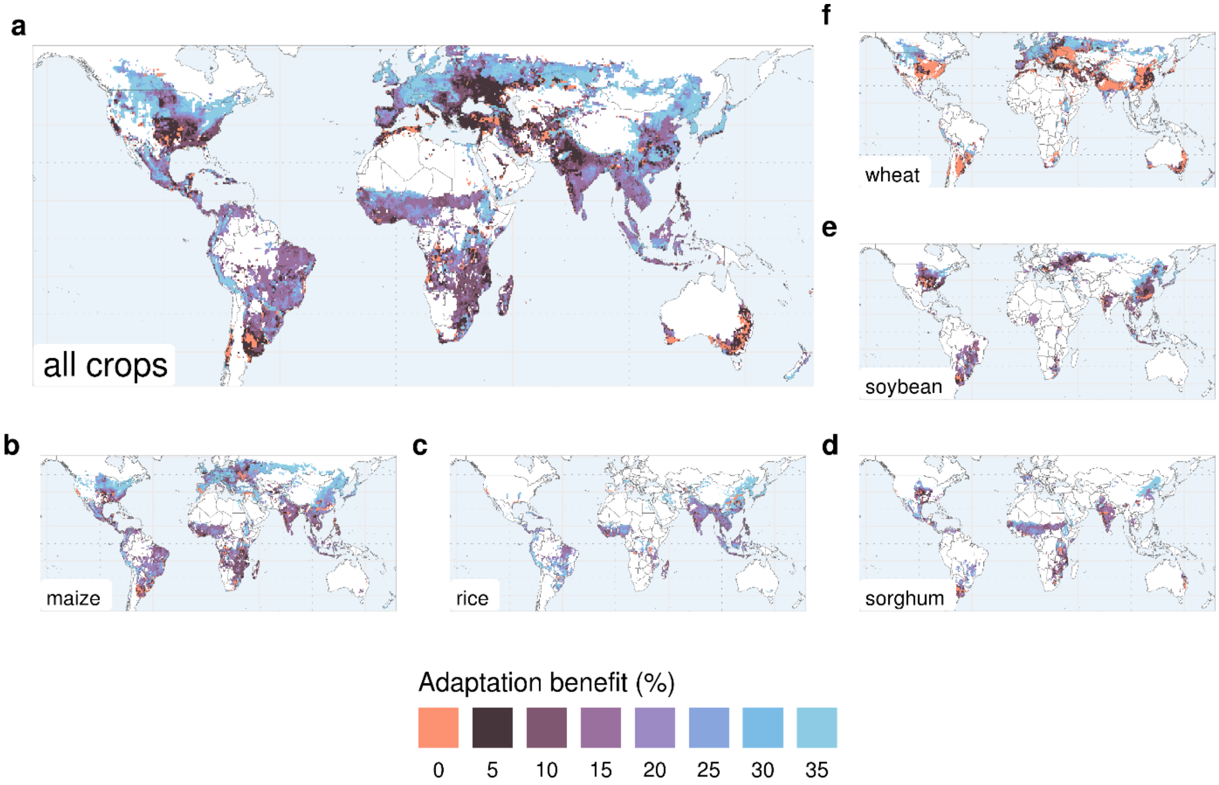

Supplementary Figure 17. Geographic patterns of crop yield benefits from sowing and cultivar adaptation. Yield benefits are computed as the relative difference (%) between the *timely adaptation* and *no adaptation* scenarios in the same climate period (2080-2099, RCP6.0, HadGEM2-ES). No-adaptation indicates a scenario in which crop sowing dates and cultivars remain unchanged compared to the reference period (1986-2005). Yield benefits are reported for (A) all five crops (maize, rice, soybean, sorghum and wheat) aggregated (area-weighted mean of yields per grid cell) and for (B-F) each individual crop. Adaptation benefits are shown as the mean across four GCMs. In grid cells where adaptation lead to either no benefit or maladaptation (negative yield change) adaptation is not considered.

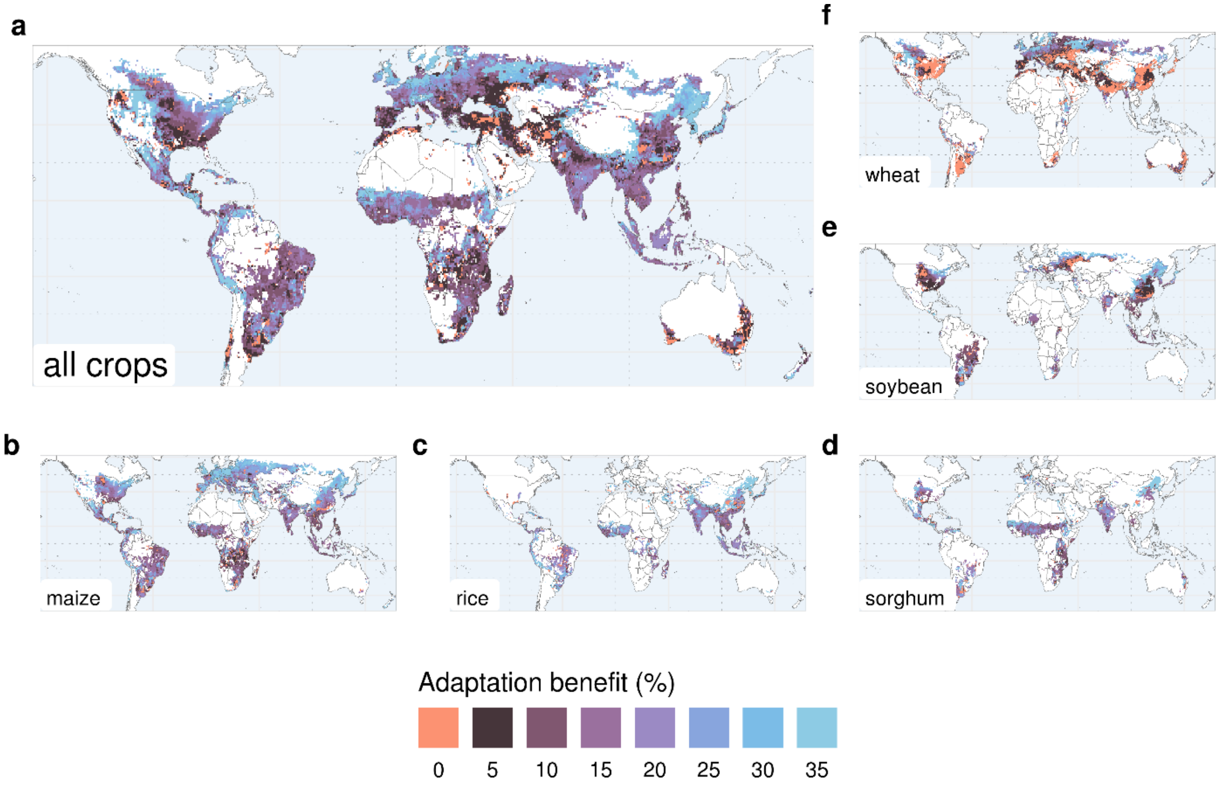

Supplementary Figure 18. Geographic patterns of crop yield benefits from sowing and cultivar adaptation. Yield benefits are computed as the relative difference (%) between the *timely adaptation* and *no adaptation* scenarios in the same climate period (2080-2099, RCP6.0, IPSL-CM5A-LR). No-adaptation indicates a scenario in which crop sowing dates and cultivars remain unchanged compared to the reference period (1986-2005). Yield benefits are reported for (A) all five crops (maize, rice, soybean, sorghum and wheat) aggregated (area-weighted mean of yields per grid cell) and for (B-F) each individual crop. Adaptation benefits are shown as the mean across four GCMs. In grid cells where adaptation lead to either no benefit or maladaptation (negative yield change) adaptation is not considered.

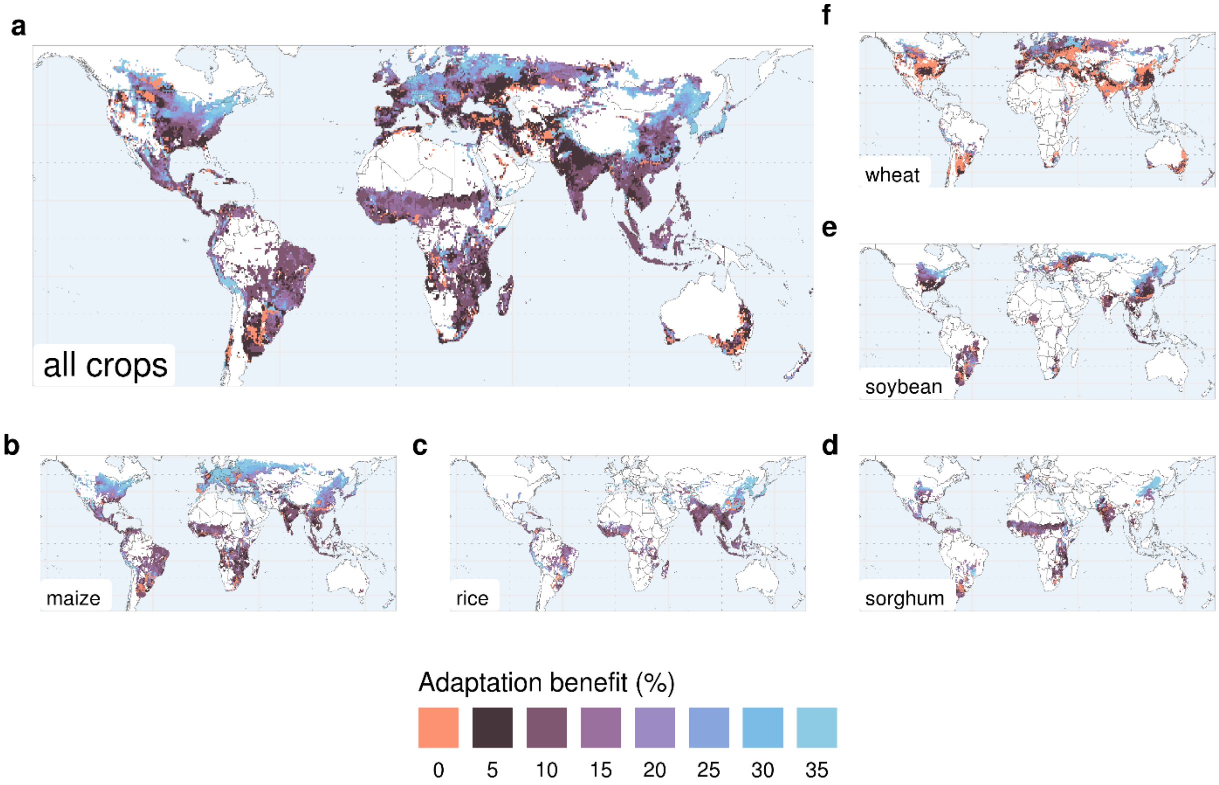

Supplementary Figure 19. Geographic patterns of crop yield benefits from sowing and cultivar adaptation. Yield benefits are computed as the relative difference (%) between the *timely adaptation* and *no adaptation* scenarios in the same climate period (2080-2099, RCP6.0, MIROC5). No-adaptation indicates a scenario in which crop sowing dates and cultivars remain unchanged compared to the reference period (1986-2005). Yield benefits are reported for (A) all five crops (maize, rice, soybean, sorghum and wheat) aggregated (area-weighted mean of yields per grid cell) and for (B-F) each individual crop. Adaptation benefits are shown as the mean across four GCMs. In grid cells where adaptation lead to either no benefit or maladaptation (negative yield change) adaptation is not considered.

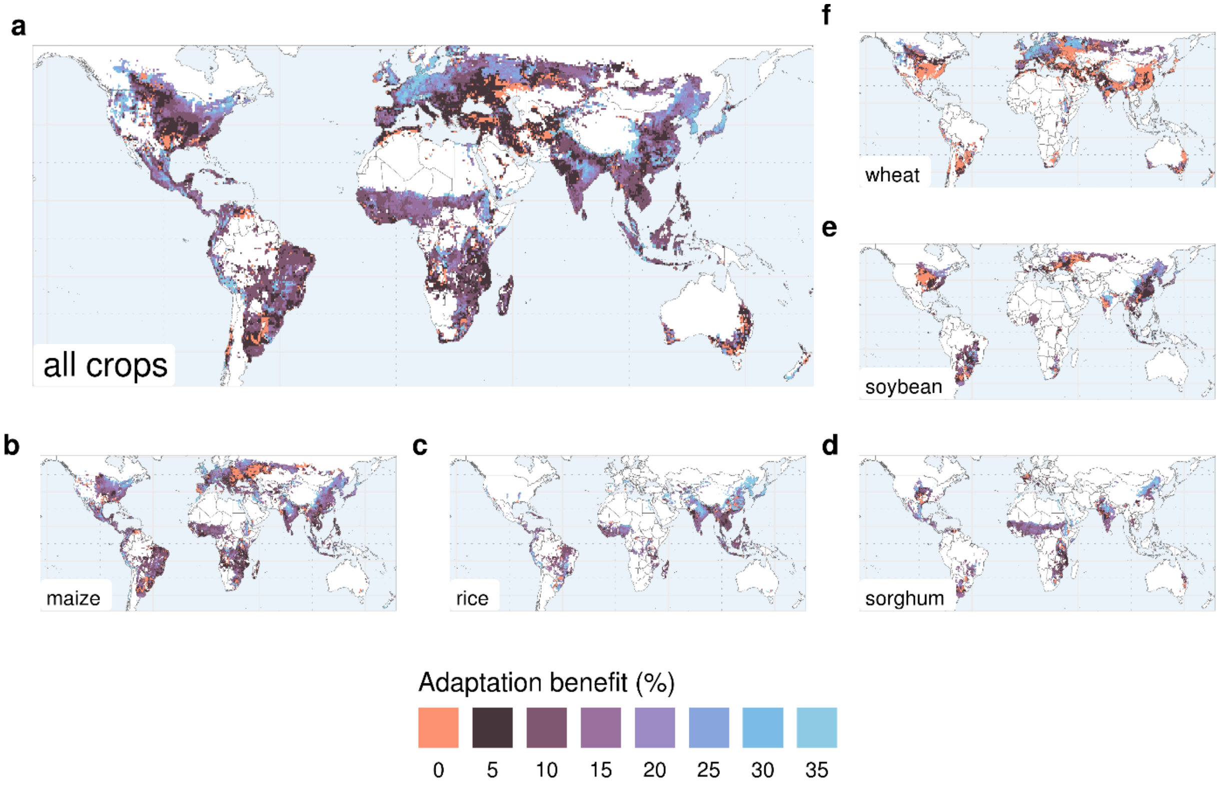

Supplementary Figure 20. Geographic patterns of crop yield benefits from sowing and cultivar adaptation. Yield benefits are computed as the relative difference (%) between the *timely adaptation* and *no adaptation* scenarios in the same climate period (2080-2099, RCP6.0, GFDL-ESM2M). No-adaptation indicates a scenario in which crop sowing dates and cultivars remain unchanged compared to the reference period (1986-2005). Yield benefits are reported for (A) all five crops (maize, rice, soybean, sorghum and wheat) aggregated (area-weighted mean of yields per grid cell) and for (B-F) each individual crop. Adaptation benefits are shown as the mean across four GCMs. In grid cells where adaptation lead to either no benefit or maladaptation (negative yield change) adaptation is not considered.

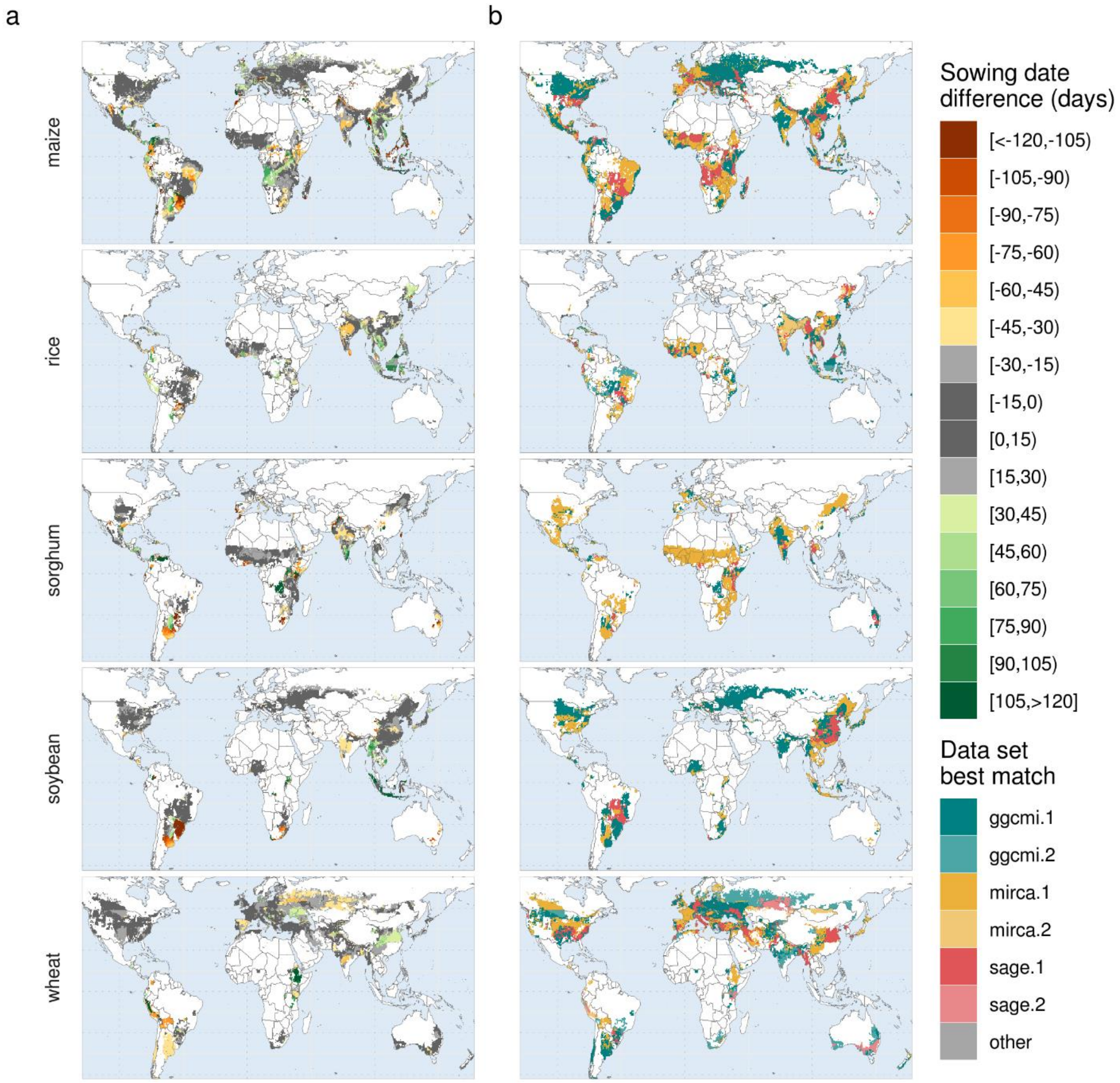

Supplementary Figure 21. Sowing dates evaluation for rainfed crops. (A) Grid-cell level comparison of simulated and observed sowing dates (day of the year) for five rainfed crops. The color scale indicates the differences (days) between simulated and observed dates. (B) Observational dataset used for the comparison in each grid cell. If for a grid cell, more than one data source is available, we select the one with the least deviation in total growing season form the simulated dates. The least deviation is calculated as the sum of absolute sowing dates difference and the absolute maturity dates difference ($|sowing_{sim} - sowing_{obs}| + |maturity_{sim} - maturity_{obs}|$). Each point represents a country and a crop and the size of the points represents the area of that country. The area-weighted Mean Absolute Error (MAE) is reported for each crop. Average crop calendars simulated by the rule-base model have been driven by observation-based climate (WFDEI[3]) for the period 1979-2012. Observed dataset include different sources[4–6] from which the best matching season with simulated dates have been selected.

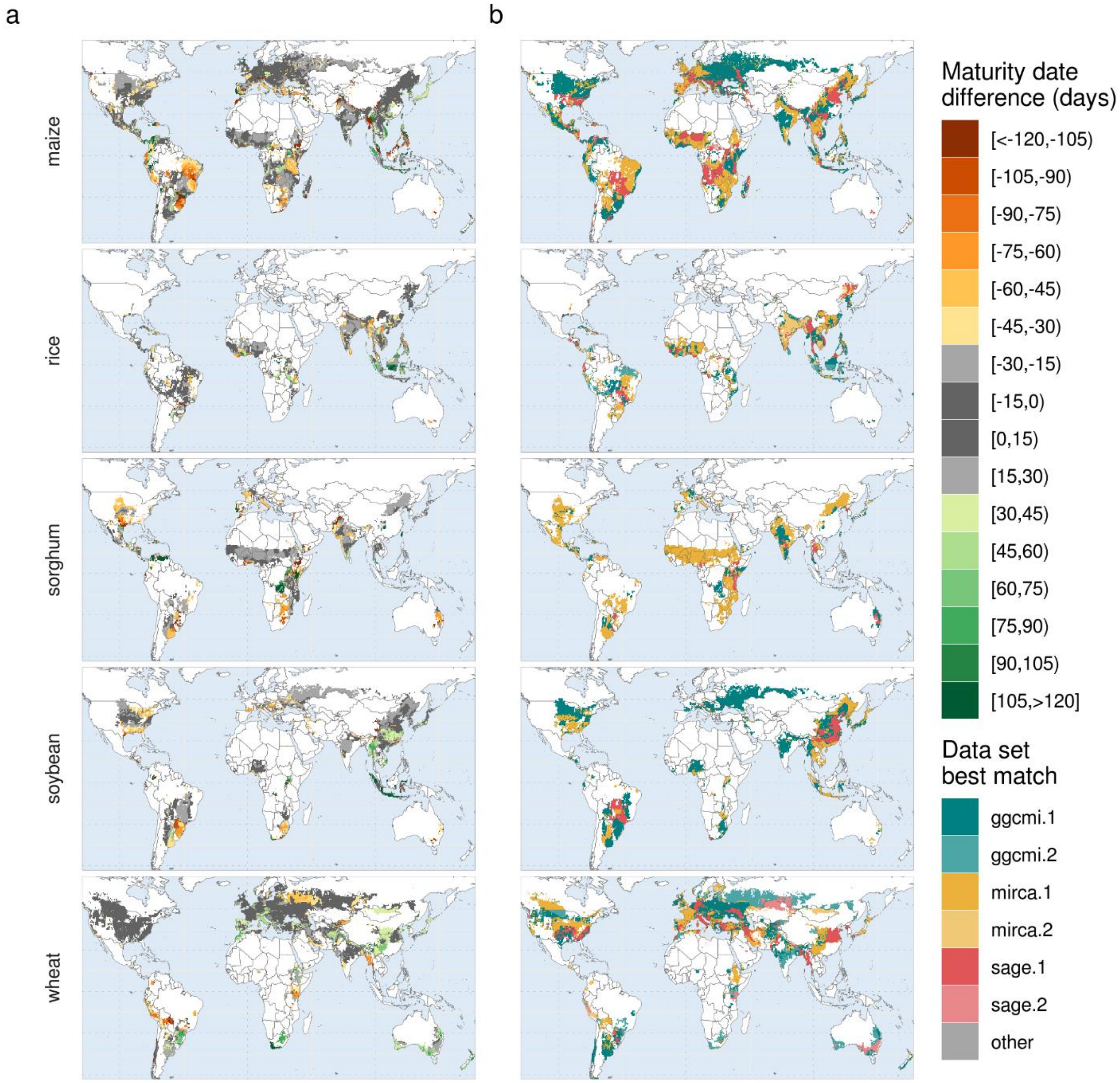

Supplementary Figure 22. Maturity dates evaluation for rainfed crops. (A) Grid-cell level comparison of simulated and observed maturity dates (day of the year) for five rainfed crops. The color scale indicates the differences (days) between simulated and observed dates. (B) Observational dataset used for the comparison in each grid cell. If for a grid cell, more than one data source is available, we select the one with the least deviation in total growing season form the simulated dates. The least deviation is calculated as the sum of absolute sowing dates difference and the absolute maturity dates difference ($|sowing_{sim} - sowing_{obs}| + |maturity_{sim} - maturity_{obs}|$). Each point represents a country and a crop and the size of the points represents the area of that country. The area-weighted Mean Absolute Error (MAE) is reported for each crop. Average crop calendars simulated by the rule-base model have been driven by observation-based climate (WFDEI[3]) for the period 1979-2012. Observed dataset include different sources[4–6] from which the best matching season with simulated dates have been selected.

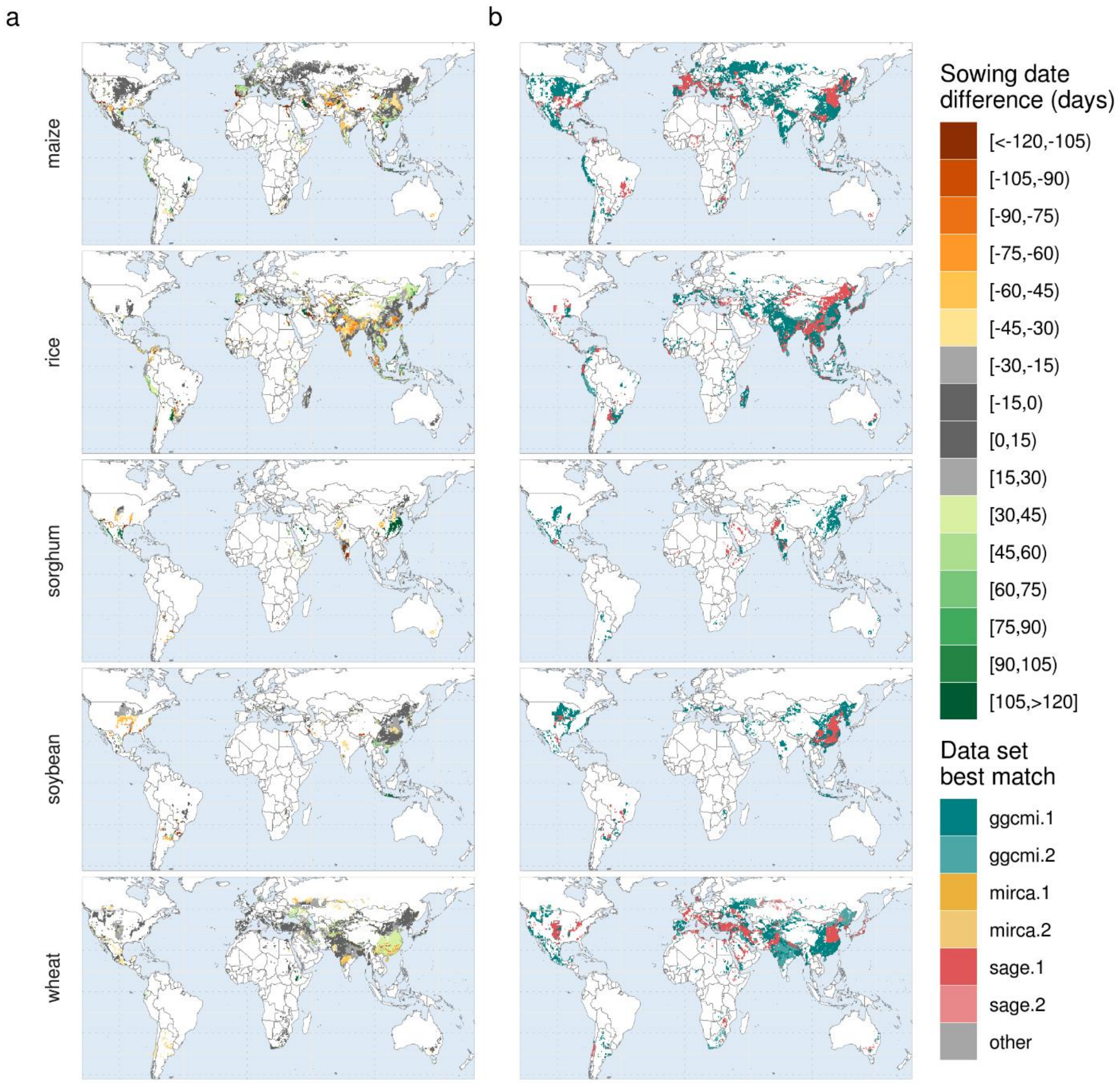

Supplementary Figure 23. Sowing dates evaluation for irrigated crops. (A) Grid-cell level comparison of simulated and observed sowing dates (day of the year) for five irrigated crops. The color scale indicates the differences (days) between simulated and observed dates. (B) Observational dataset used for the comparison in each grid cell. If for a grid cell, more than one data source is available, we select the one with the least deviation in total growing season form the simulated dates. The least deviation is calculated as the sum of absolute sowing dates difference and the absolute maturity dates difference ($|sowing_{sim} - sowing_{obs}| + |maturity_{sim} - maturity_{obs}|$). Each point represents a country and a crop and the size of the points represents the area of that country. The area-weighted Mean Absolute Error (MAE) is reported for each crop. Average crop calendars simulated by the rule-base model have been driven by observation-based climate (WFDEI[3]) for the period 1979-2012. Observed dataset include different sources[4–6] from which the best matching season with simulated dates have been selected.

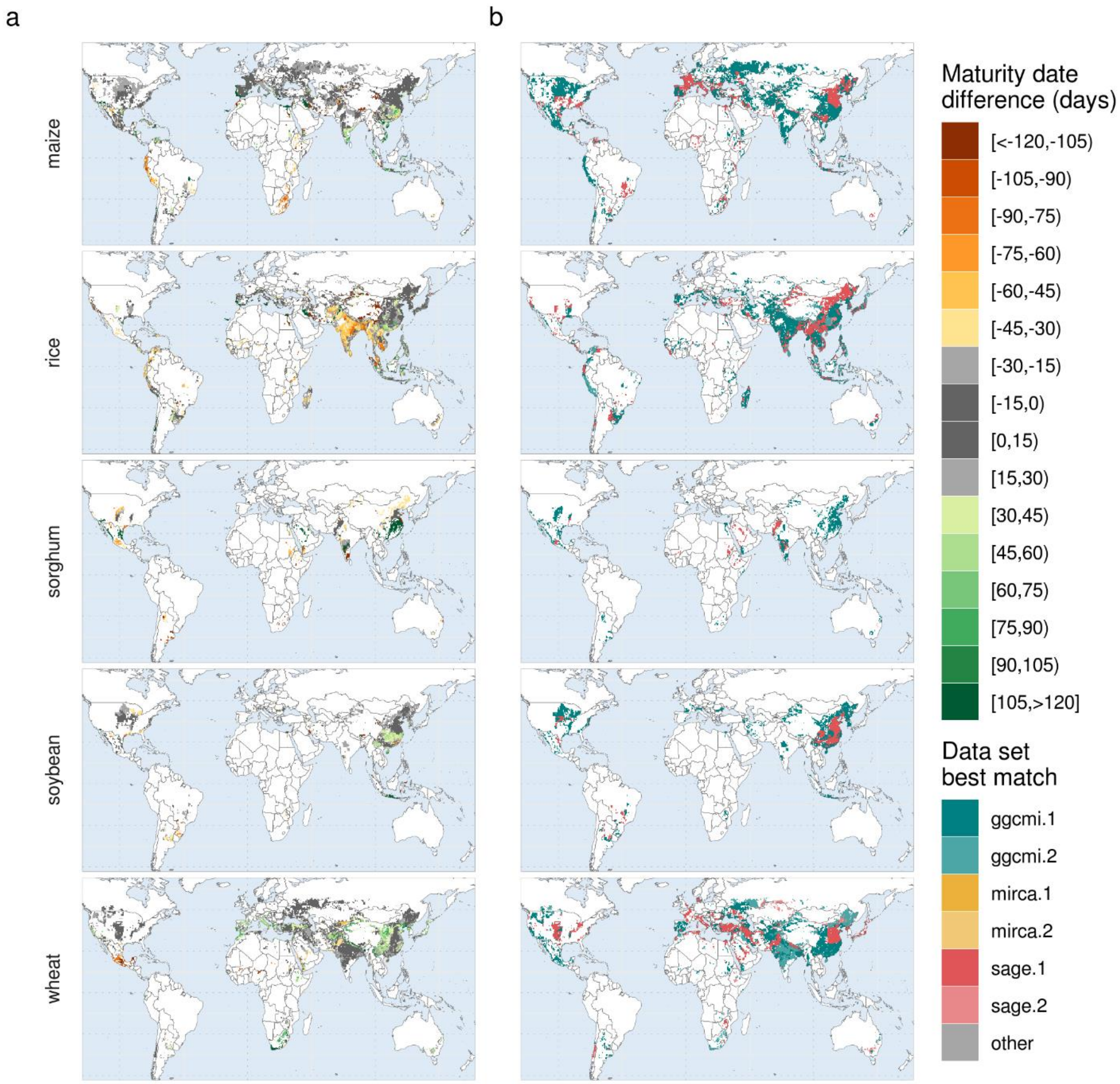

Supplementary Figure 24. Maturity dates evaluation for irrigated crops. (A) Grid-cell level comparison of simulated and observed maturity dates (day of the year) for five irrigated crops. The color scale indicates the differences (days) between simulated and observed dates. (B) Observational dataset used for the comparison in each grid cell. If for a grid cell, more than one data source is available, we select the one with the least deviation in total growing season form the simulated dates. The least deviation is calculated as the sum of absolute sowing dates difference and the absolute maturity dates difference ($|sowing_{sim} - sowing_{obs}| + |maturity_{sim} - maturity_{obs}|$). Each point represents a country and a crop and the size of the points represents the area of that country. The area-weighted Mean Absolute Error (MAE) is reported for each crop. Average crop calendars simulated by the rule-base model have been driven by observation-based climate (WFDEI[3]) for the period 1979-2012. Observed dataset include different sources[4–6] from which the best matching season with simulated dates have been selected.

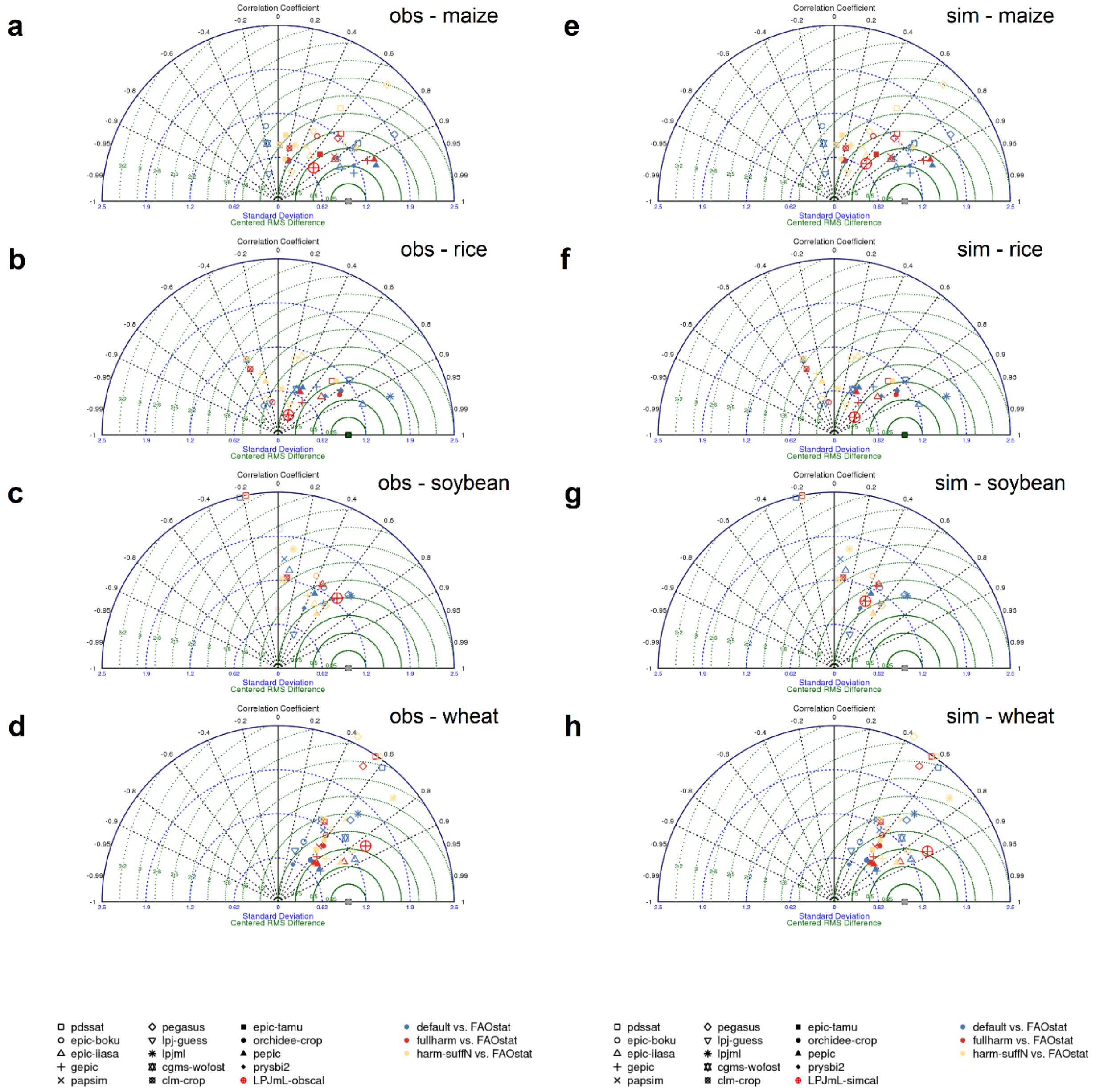

Supplementary Figure 25. Crop yield evaluation. Taylor diagram showing simulated yields (from LPJmL crop model) agreement with observed yields (FAO) at the country-scale. Performances are shown for two sets of simulations, differing for the crop calendar dataset used to constrain crop phenology: (A-D) Observation-based (E-H) rule-based. The red crossed circle indicates the performance of the runs described in this paper. The other symbols represent published benchmark model outputs from a large set of global gridded crop models[7]. Taylor diagrams were generated with the GGCMI online evaluation tool publicly available at https://mygeohub.org/resources/agmip. Full set of evaluation results are reported as separated Supplementary Materials.

Supplementary Table 1. Temperature thresholds used for computing the cropping calendars. Values are taken from [2,8]. T_{base} *sowing* is the temperature threshold for sowing; T_{base} *yield formation* is the base temperature for the crop reproductive development, below which the crop cannot complete the reproductive cycle; T_{opt} *yield formation* is the optimum temperature for the crop reproductive grain formation; T_{base} *thermal time* is the base temperature for phenological development.

Crop	T_{base} *sowing (°C)*	T_{base} *yield formation (°C)*	T_{opt} *yield formation(°C)*	T_{base} *thermal time (°C)*
maize	14	7	30	6
rice	18	8	24	8
sorghum	12	8	25	8
soybean	13	6	23	7
spring wheat	5	1	25	0
winter wheat	12	1	25	0

Supplementary Table 2. Crop calendars evaluation. Area-weighted Mean Absolute Error (MAE) (days) of simulated crop calendars for each crop and irrigation setting. Average crop calendars simulated by the rule-base model have been driven by observation-based climate (WFDEI[3]) for the period 1979-2012. Observed dataset include different sources[4–6] from which the best matching season with simulated dates have been selected.

Crop	*Irrigation*	*MAE sowing (days)*	*MAE maturity (days)*	*MAE grow. period (days)*
maize	rainfed	29	32	19
maize	irrigated	30	22	26
rice	rainfed	41	25	33
rice	irrigated	34	39	26
sorghum	rainfed	28	27	13
sorghum	irrigated	74	80	30
soybean	rainfed	43	49	26
soybean	irrigated	29	20	31
wheat	rainfed	19	50	26
wheat	irrigated	19	19	24

Supplementary Table 3. Climate change yield impacts from a global model ensemble. Projected changes in global crop yields (2069–2099 compared to 1983–2013) simulated by a large ensemble of Global Gridded Crop Models from the AgMIP-GGCMI project[6], based on CMIP6, RCP8.5 (SSP5) and RCP2.6 (SSP1). Although not directly comparable to our study (we use CMIP5, RCP6.0), provide context showing the mean, range and standard deviation (SD) of crop responses to CO_2 increase and climate change from state-of-the-art global-scale crop models.

Crop	*RCP*	*Mean*	*Min*	*Max*	*SD*	*LPJmL*
maize	8.5	-24.1	-60.0	4.6	19.2	-7.4
rice	8.5	1.7	-27.0	28.4	17.4	18.3
soybean	8.5	-2.1	-63.5	39.4	26.7	24.4
wheat	8.5	17.5	-22.5	55.8	25.3	15.1
maize	2.6	-6.4	-19.1	1.0	6.5	-0.3
rice	2.6	3.4	-6.0	12.4	5.6	2.8
soybean	2.6	2.0	-18.0	13.7	8.1	8.2
wheat	2.6	8.8	-0.3	27.5	8.6	6.0

Supplementary References

1. Frieler, K. *et al.* Assessing the impacts of 1.5°C global warming – simulation protocol of the Inter-Sectoral Impact Model Intercomparison Project (ISIMIP2b). *Geoscientific Model Development* **10**, 4321–4345 (2017).
2. Waha, K., Bussel, L. G. J. van, Müller, C. & Bondeau, A. Climate-driven simulation of global crop sowing dates. *Global Ecology and Biogeography* **21**, 247–259 (2012).
3. Weedon, G. P. *et al.* The WFDEI meteorological forcing data set: WATCH Forcing Data methodology applied to ERA-Interim reanalysis data. *Water Resources Research* **50**, 7505–7514 (2014).
4. Portmann, F. T., Siebert, S. & Döll, P. MIRCA2000-Global monthly irrigated and rainfed crop areas around the year 2000: A new high-resolution data set for agricultural and hydrological modeling. *Global Biogeochemical Cycles* **24**, GB1011 (2010).
5. Sacks, W. J., Deryng, D., Foley, J. A. & Ramankutty, N. Crop planting dates: an analysis of global patterns. *Global Ecology and Biogeography* **19**, 607–620 (2010).
6. Jägermeyr, J. *et al.* Climate impacts on global agriculture emerge earlier in new generation of climate and crop models. *Nature Food* **2**, 873–885 (2021).
7. Müller, C. *et al.* Global gridded crop model evaluation: benchmarking, skills, deficiencies and implications. *Geoscientific Model Development* **10**, 1403–1422 (2017).
8. Minoli, S., Egli, D. B., Rolinski, S. & Müller, C. Modelling cropping periods of grain crops at the global scale. *Global and Planetary Change* **174**, 35–46 (2019).
