# Supplementary material for: Global crop yields can be lifted by timely adaptation of growing periods to climate change
Source: Nat Commun. 2022 Nov 18;13:7079. doi: 10.1038/s41467-022-34411-5 (PMC9674574; doi:10.1038/s41467-022-34411-5)
Supplement: Supplementary file 2 — Reporting Summary [file 41467_2022_34411_MOESM2_ESM.pdf]

nature portfolio

Corresponding author(s): Sara Minoli

Last updated by author(s): Sep 8, 2022

Reporting Summary

Nature Portfolio wishes to improve the reproducibility of the work that we publish. This form provides structure for consistency and transparency in reporting. For further information on Nature Portfolio policies, see our Editorial Policies and the Editorial Policy Checklist.

Statistics

For all statistical analyses, confirm that the following items are present in the figure legend, table legend, main text, or Methods section.

n/a	Confirmed	
☐	☒	The exact sample size (*n*) for each experimental group/condition, given as a discrete number and unit of measurement
☒	☐	A statement on whether measurements were taken from distinct samples or whether the same sample was measured repeatedly
☒	☐	The statistical test(s) used AND whether they are one- or two-sided<br>*Only common tests should be described solely by name; describe more complex techniques in the Methods section.*
☐	☒	A description of all covariates tested
☒	☐	A description of any assumptions or corrections, such as tests of normality and adjustment for multiple comparisons
☐	☒	A full description of the statistical parameters including central tendency (e.g. means) or other basic estimates (e.g. regression coefficient) AND variation (e.g. standard deviation) or associated estimates of uncertainty (e.g. confidence intervals)
☒	☐	For null hypothesis testing, the test statistic (e.g. *F*, *t*, *r*) with confidence intervals, effect sizes, degrees of freedom and *P* value noted<br>*Give P values as exact values whenever suitable.*
☒	☐	For Bayesian analysis, information on the choice of priors and Markov chain Monte Carlo settings
☒	☐	For hierarchical and complex designs, identification of the appropriate level for tests and full reporting of outcomes
☒	☐	Estimates of effect sizes (e.g. Cohen's *d*, Pearson's *r*), indicating how they were calculated

Our web collection on statistics for biologists contains articles on many of the points above.

Software and code

Policy information about availability of computer code

Data collection	- The crop calendar dataset was generated by a custom code implemented in R for the purpose of this study, following previously published approaches (Waha et al., 2012; Minoli et al., 2019). An updated version of the crop calendar model in form of an R package (v0.1.0) is available on GitHub at https://github.com/AgMIP-GGCMI/cropCalendars.<br>- Crop productivity estimates were generated by the global gridded crop model LPJmL5.0-gsadapt (Bloh et al., 2018; Lutz et al., 2019). Modifications of the crop phenological module in the LPJmL model are described in the methods.<br>- The MADRaT tool v0.0.2 (Dietrich et al., 2020) was used for dis-aggregating and re-mapping the LUH2v2 landuse dataset.<br>- All codes used in the study are made available alongside the manuscript at the Zenodo public repository (DOI: 10.5281/zenodo.7038163).
Data analysis	- For data processing and analysis we used R 2021 (v4.0.5).<br>- We evaluate the LPJmL model outputs against a standard benchmark for global gridded crop models evaluation (v1.3), which is available online (https://mygeohub.org/resources/agmip).

For manuscripts utilizing custom algorithms or software that are central to the research but not yet described in published literature, software must be made available to editors and reviewers. We strongly encourage code deposition in a community repository (e.g. GitHub). See the Nature Portfolio guidelines for submitting code & software for further information.

Data

Policy information about availability of data

All manuscripts must include a data availability statement. This statement should provide the following information, where applicable:

- Accession codes, unique identifiers, or web links for publicly available datasets
- A description of any restrictions on data availability
- For clinical datasets or third party data, please ensure that the statement adheres to our policy

- The source code of the crop calendar model; the source code of the LPJmL model ; the scripts for data analysis; the LPJmL model raw outputs; and the results of the GGCMI crop model evaluation tools are made publicly available on Zenodo (DOI: 10.5281/zenodo.7038163).
- Climate reanalysis data (WFDEI) and model projection of the four GCMs (HadGEM2-ES, GFDL-ESM2M, IPSL-CM5A-LR, MIROC5) are available at https://www.isimip.org/.
- All other input data necessary to run the LPJmL model can be provided upon request to the corresponding author.

Human research participants

Policy information about studies involving human research participants and Sex and Gender in Research.

Reporting on sex and gender	N/A
Population characteristics	N/A
Recruitment	N/A
Ethics oversight	N/A

Note that full information on the approval of the study protocol must also be provided in the manuscript.

Field-specific reporting

Please select the one below that is the best fit for your research. If you are not sure, read the appropriate sections before making your selection.

☐ Life sciences ☐ Behavioural & social sciences ☒ Ecological, evolutionary & environmental sciences

For a reference copy of the document with all sections, see nature.com/documents/nr-reporting-summary-flat.pdf

Ecological, evolutionary & environmental sciences study design

All studies must disclose on these points even when the disclosure is negative.

Study description	We present a crop-model based quantitative study of the effect of farmers adaptive management under climate change on crop growing periods and productivity. We conduct a scenario-based simulation experiment including three levels of adaptation, four General Circulation Models, two CO2 levels, five different crop types and two irrigation levels. For each scenario, we conduct gridded simulations covering the current extent of the global cropland area.
Research sample	- Five crops (maize, rice, sorghum, soybean and wheat)<br>- Four climate scenarios from the Coupled Model Intercomparison Project (CMIP) phase 5
Sampling strategy	- We have investigated adaptation of the top five staple crops that are globally relevant in terms of cultivated area and global production and therefore of importance for food security and land-use change implications across all continents.<br>- We have run simulations under four climate scenarios from the Coupled Model Intercomparison Project (CMIP) phase 5 as selected by the ISIMIP project, which are high-standard bias-adjusted products, widely used in the climate impact assessment community.
Data collection	SM performed the model simulations and collected the outputs of the crop-calendar model (average sowing dates, maturity dates, thermal unit requirements) and of the LPJmL model (annual maturity dates and yields).
Timing and spatial scale	Our analysis is conducted at the global scale covering the entire current cropland of the considered crops (maize, rice, sorghum, soybean, wheat). All input and outputs datasets are gridded at 0.5 degrees spatial resolution. Our analysis focus on two climatic periods, historical (1986-2005) and end-of-century (2080-2099) climate, for which annual outputs of maturity dates and yields produced.
Data exclusions	No data were excluded from the analysis.
Reproducibility	To allow full reproducibility, all simulations, data analysis and result visualization are script-based, complemented by configuration files and detailed "readme" files.

Randomization	The study did not require randomization, as the simulation models used are deterministic and the analysis based on counterfactual-scenarios, implying that each adaptation scenario has been simulated for each crop and climate projection over the exact same simulation domain (i.e. grid cells and years).
Blinding	Blinding was not applicable due to the modeling nature of this study, which requires model inputs and outputs to be preprocessed and displayed for identifying technical issues. The scenario experimental design and the non-exclusion of any data ensures the least bias in result interpretation.

Did the study involve field work? ☐ Yes ☒ No

Reporting for specific materials, systems and methods

We require information from authors about some types of materials, experimental systems and methods used in many studies. Here, indicate whether each material, system or method listed is relevant to your study. If you are not sure if a list item applies to your research, read the appropriate section before selecting a response.

Materials & experimental systems

n/a	Involved in the study
☒	☐ Antibodies
☒	☐ Eukaryotic cell lines
☒	☐ Palaeontology and archaeology
☒	☐ Animals and other organisms
☒	☐ Clinical data
☒	☐ Dual use research of concern

Methods

n/a	Involved in the study
☒	☐ ChIP-seq
☒	☐ Flow cytometry
☒	☐ MRI-based neuroimaging
